# Relationship between gait parameters and cognitive indexes in adult aging

**DOI:** 10.1371/journal.pone.0291963

**Published:** 2023-09-21

**Authors:** Tania Aznielle-Rodríguez, Lídice Galán-García, Marlis Ontivero-Ortega, Karen Aguilar-Mateu, Ana M. Castro-Laguardia, Ana Fernández-Nin, Daysi García-Agustín, Mitchell Valdés-Sosa

**Affiliations:** 1 Department of Electronics, Cuban Center for Neuroscience, Havana, Cuba; 2 Department of Neuroinformatics, Cuban Center for Neuroscience, Havana, Cuba; 3 Department of Data Analysis, Faculty of Psychological and Educational Sciences, Ghent University, Ghent, Belgium; 4 Department of Cognitive Neuroscience, Cuban Center for Neuroscience, Havana, Cuba; 5 Centro de Investigaciones Sobre Longevidad, Envejecimiento y Salud, CITED, Havana, Cuba; Public Library of Science, UNITED KINGDOM

## Abstract

**Purpose:**

This study aimed to identify the most effective summary cognitive index predicted from spatio-temporal gait features (STGF) extracted from gait patterns.

**Methods:**

The study involved 125 participants, including 40 young (mean age: 27.65 years, 50% women), and 85 older adults (mean age: 73.25 years, 62.35% women). The group of older adults included both healthy adults and those with Mild Cognitive Impairment (MCI). Participant´s performance in various cognitive domains was evaluated using 12 cognitive measures from five neuropsychological tests. Four summary cognitive indexes were calculated for each case: 1) the z-score of Mini-Mental State Examination (MMSE) from a population norm (MMSE z-score); 2) the sum of the absolute z-scores of the patients’ neuropsychological measures from a population norm (ZSum); 3) the first principal component scores obtained from the individual cognitive variables z-scores (PCCog); and 4) the Mahalanobis distance between the vector that represents the subject’s cognitive state (defined by the 12 cognitive variables) and the vector corresponding to a population norm (MDCog). The gait patterns were recorded using a body-fixed Inertial Measurement Unit while participants executed four walking tasks (normal, fast, easy- and hard-dual tasks). Sixteen STGF for each walking task, and the dual-task costs for the dual tasks (when a subject performs an attention-demanding task and walks at the same time) were computed. After applied Principal Component Analysis to gait measures (96 features), a robust regression was used to predict each cognitive index and individual cognitive variable. The adjusted proportion of variance (adjusted-R^2^) coefficients were reported, and confidence intervals were estimated using the bootstrap procedure.

**Results:**

The mean values of adjusted-R^2^ for the summary cognitive indexes were as follows: 0.0248 for MMSE z-score, 0.0080 for ZSum, 0.0033 for PCCog, and 0.4445 for MDCog. The mean adjusted-R^2^ values for the z-scores of individual cognitive variables ranged between 0.0009 and 0.0693. Multiple linear regression was only statistically significant for MDCog, with the highest estimated adjusted-R^2^ value.

**Conclusions:**

The association between individual cognitive variables and most of the summary cognitive indexes with gait parameters was weak. However, the MDCog index showed a stronger and significant association with the STGF, exhibiting the highest value of the proportion of the variance that can be explained by the predictor variables. These findings suggest that the MDCog index may be a useful tool in studying the relationship between gait patterns and cognition.

## Introduction

The use of wearable sensors, such as Inertial Measurement Units (IMU), has made it possible to record gait patterns and extract spatio-temporal gait features (STGF) even outside of specialized laboratories [1]. This has facilitated studies of the relationship between gait disturbances and cognitive impairment within the community, particularly in relation to aging [2–4]. Overall findings indicate that gait quality deteriorates with age [4], especially in the presence of cognitive impairment [2–5]. However, the results are not consistently conclusive and are challenging to generalize to new subject samples [3]. This may be attributed to several issues.

The first issue is the diversity of cognitive variables used in studies to assess the gait-cognition relationship. Some studies have used scores obtained from rapid clinical tools that provide a global assessment of cognition, e.g. the Mini-Mental State Examination (MMSE) [6] or the Montreal Cognitive Assessment (MoCA) [7]. However, these instruments are semi-quantitative and have low sensitivity and specificity in detecting cognitive impairment (MMSE Sensitivity: 53.1% [8] and 62.7% [9]; MMSE Specificity: 63.3% [9]). MMSE primarily focus on language and memory domains, neglecting other important cognitive domains for gait quality, such as processing speed [3]. It is useful in advanced stages of cognitive decline, while MoCA is sensitive in Mild Cognitive Impairment (MCI) [7].

Other studies have used specific cognitive variables obtained from various neuropsychological tests [10–13], as highlighted in the review by Demnitz et al. [3]. These tests assess cognitive domains such as attention, executive function, memory, visuospatial function, processing speed, among others. Some studies have explored the combination of multiple cognitive variables into a single measure [8, 14–16]. For example, MacAulay et al. [8] computed an index using the z-scores derived from executive attention and visuospatial function scores (domains highly related to gait) to differentiate between healthy older adults and those with cognitive impairment. This index showed a good correlation with gait speed, the only STGF analyzed, but other cognitive domains were not considered. It is necessary to identify a summary measure of cognition that encompasses a broader range of cognitive domains, accurately reflects a subject’s cognitive state, and exhibits a high correlation with gait features.

The second issue is that few studies have used normative data to correct for age-related changes in cognitive test scores, which is crucial for assessing the true severity of cognitive impairments (with exceptions [15]). Consequently, the extent to which a participant’s cognitive functions deviate from the population mean is generally not reported in studies investigating the relationship between cognition and gait.

Thirdly, many studies have involved a limited number of participants, hinder the reproducibility of findings due to low statistical power [8, 17, 18]. Previous studies have included 65 [8], 56 [18] and 15 [17] participants. Typically, studies focus on gait and cognition in the context of aging, comprising healthy older adults [18], older adults with MCI [17], or a combination of both groups [8]. Furthermore, only a few STGF have been examined, with some studies considering only one feature, such as gait speed [3].

The present study aimed to address the difficulties mentioned above by proposing four cognitive indexes to characterize the participants’ cognitive status. These indexes are derived from five cognitive tests that evaluate various cognitive domains. One of them uses the scores of the first principal component obtained through Principal Component Analysis (PCA) to reduce dimensionality and redundancy in cognitive data [19]. Another index reflects the deviation of a subject’s results from the normal profile of the population, using the Mahalanobis distance. This statistical tool preserves the quantitative aspect of neuropsychological tests and considers correlations between variables to prevent spurious inflation of the estimated distance. It has previously been used to measure the deviation of a subject’s cognitive profile from the average profile of the population [20, 21]. In this study, normative data for the Cuban population in the five cognitive tests were utilized. The sample included 125 participants across a wide age range, encompassing both young and older adults. To evaluate the strength of the association between the four summary cognitive indexes and a set of STGF extracted from gait patterns recorded during different walking tasks, multiple linear regressions were used. The primary goal was to identify the summary cognitive index best predicted from STGF, considering that gait analysis is easy to perform, offers quantitative measurements and could potentially serve as a rapid screening and longitudinal assessment tool.

## Methods

### Participants

Approximately 200 participants from various health institutions in Havana city and from the Cuban Center for Neuroscience (CNEURO) were screened for inclusion in the study. This sample was previously used in a prior paper [21], and details regarding its origin can be found in S1 Table in S1 File. The recruitment process is described in detail in the Supporting information. Two questionnaires were administered: 1) to determine the Katz Index of independence [22, 23], and 2) for a general clinical evaluation, including pathological history, associated comorbidities, toxic habits, as well as gait problems. All participants underwent a neurological and physical examination and a cognitive assessment before performing the walking tasks.

**Fig 1:**
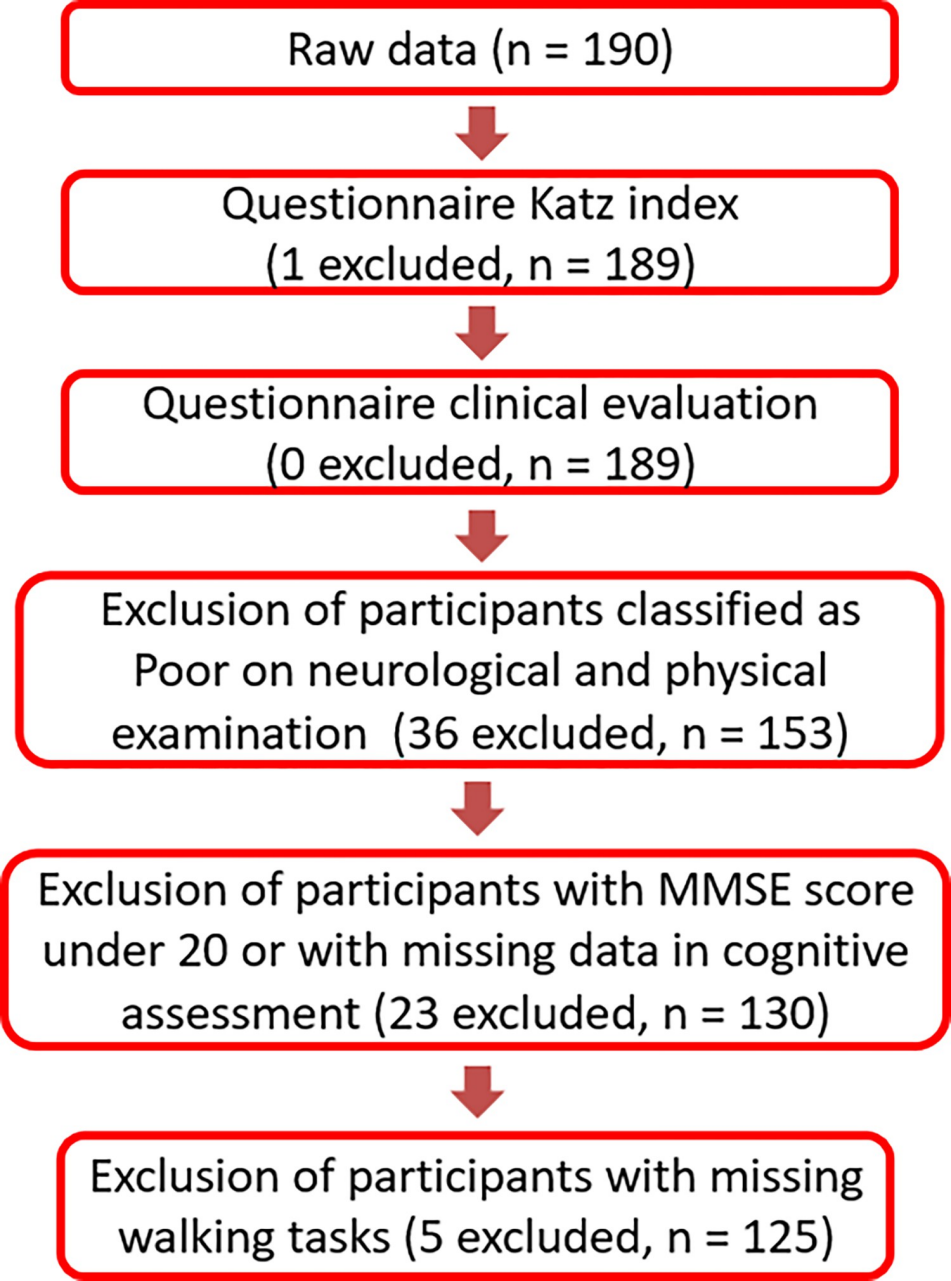
Flowchart of participant´s selection. (n: sample size).

The inclusion criteria were the participant’s agreement, young adults aged between 20 and 40 years and older adults ages above 60 years, and a Katz Index of independence ≥ 4, indicating functional independence without the need for supervision or external assistance in performing basic daily activities [22, 23]. Participants with an inability to walk, major neurological disorders, musculoskeletal system diseases, or severe cognitive impairment were excluded. The flowchart of participant´s selection is shown in Fig 1.

Written informed consent was obtained from all the participants and doctors in the health institutions. The study was approved by the ethics committee of CNEURO in compliance with the Helsinki declaration.

### Experiment

Participants were instructed to walk a distance of 40 m (20 m in each direction) in a flat and obstacle-free environment (see S1 Fig in S1 File) while performing four different walking tasks: 1) walking freely at a self-chosen comfortable speed (NormalW); 2) walking at a self-chosen comfortable speed while counting their steps, an easy cognitive task (EasyD); 3) walking at a self-chosen comfortable speed while simultaneously counting backward from 100, a hard cognitive task (HardD); and 4) walking as fast as possible without running (FastW). During tasks 2 and 3, subjects performed a cognitive task while walking, known as a dual task, which is commonly used to assess the relationship between gait, cognition, and risk of falls [24].

The participants´ gait patterns were recorded using an IMU (Bitalino RIoT, Plux Wireless Biosignals, Portugal), securely attached to a Velcro band, and positioned near the body’s center of mass at the L3 spinal level. The vertical acceleration signal was processed as described in a previous paper [25]. It was divided into two segments, one for each direction of walking, and the strides corresponding to the first and last three seconds of each segment were removed. Subsequently, a dynamic tilt correction and filtering were applied to eliminate noise. After computing the Initial Contact and Final Contact events of the gait cycles in each segment, 16 STGF were calculated for each walking task: 1) step time (StpT); 2) step time variability or step time coefficient of variation (StpTCoV); 3) stride time (StrT); 4) stride time variability or stride time coefficient of variation (StrTCoV); 5) cadence (Cd); 6) root mean square amplitude of the vertical acceleration (RMS); 7) double support duration or double support time (DSD); 8) single support duration or single support time (SSD); 9) swing duration feed 1 (SwDurF1); 10) swing duration feed 2 (SwDurF2); 11) stance duration feed 1 (StDurF1); 12) stance duration feed 2 (StDurF2); 13) step duration feed 1 (StepDurF1); 14) step duration feed 2 (StepDurF2); 15) step length (StepLg); and 16) speed (GS). All STGF were expressed in seconds (s), except Cd (steps/min), RMS (g), StepLg (m) and GS (m/s). STGF were computed using algorithms described in the literature [26–33]. The STFG calculated for the segments of the same walking task were averaged. A total of 64 STGF were obtained (16 STGF x 4 walking tasks) for each participant.

In addition to the 64 STGF, 32 dual-task costs (DTC) were calculated for the STGF in the two dual-tasks relative to the NormalW task (16 dual-task costs x 2 dual walking tasks). These costs (expressed as percentages) were calculated using Eq 1 [34].


$$DTC=\left(\frac{single\_task\_value-dual\_task\_value}{single\_task\_value}\right)*100$$ (1)

Overall, 96 measures (STGF and DTC) were obtained for each participant.

### Cognitive assessment

The cognitive abilities of the participants was assessed through five neuropsychological tests:

#### 1) Mini-Mental State Examination (MMSE)

This test uses questions to briefly explore different domains of cognition, including orientation to time and place, working memory, attention and calculation, visuospatial function, among others [6]. The score of this test (MMSE_sc) ranges from 0 to 30, and is used as a global index of cognitive function in clinical practice.

#### 2) Attentional Span or Brief Test of Attention (BTA)

This test explores attention, specifically focused on auditory divided attention [10]. It consists of two parallel forms presented audibly. In Form N, the examiner reads 10 lists of letters and numbers, and the participant must count the quantity of numbers. The participant reports the number of numbers heard during the 5 seconds after each list is read. In Form L, the target is letters instead numbers. The scores for this test are recorded as the number of correct answers for each form (variables AtentN and AtentL), with a maximum score of 10 points for each form.

#### 3) Trail Making Test (TMT)

This test evaluates attention, visuospatial abilities, mental flexibility, and executive functions [11]. It consists of two parts, A and B, each with different objectives. Part A measures the time it takes to connect consecutively numbered, semi-randomly distributed circles on a sheet of paper by drawing lines between them without lifting the pencil (variable TMT_PartA). Part B is performed on a different sheet and measures the time to connect an equal number of circles containing numbers and letters in ascending and alternating order (variable TMT_PartB).

#### 4) Hopkins Verbal Learning Test (HLVT)

This test assesses memory using six different measures [12], including immediate recognition and delayed recall. The participant is given a list of 12 semantically categorized words and must recall the list three times immediately after presentation and again after a 20-minute delay. The number of words remembered in each attempt is recorded (variables HLVT_T1, HLVT_T2 and HLVT_T3 and HLVT_TR). A yes/no recognition task is administered at the end of the test, with a list of 24 words, including the 12 words from the initial list. The number of correctly identified words and false positives for semantically related words are recorded (variables HLVT_DRCA and HLVT_DRFP, respectively).

#### 5) Digit Symbol substitution test (DS)

This test evaluates focused, selective, and sustained attention, visual perception, and processing speed [13]. The participant is required to match symbols to numbers based on a provided key located at the top of a sheet of paper. The number of correct symbol substitutions completed within 90 seconds is recorded (variable DigSim).

The twelve variables obtained from these tests are summarized in Table 1.

**Table 1:** Cognitive variables extracted from the neuropsychological tests.

Test	Domain	Cognitive variable
MMSE	Global cognition	MMSE_sc
BTA	Attentional impairment	AtentL
		AtentN
TMT	Attention, visuospatial abilities, mental flexibility and executive function	TMT_PartA
		TMT_PartB
HLVT	Memory, including immediate recognition and delayed recall	HLVT_T1
		HLVT_T2
		HLVT_T3
		HLVT_TR
		HLVT_DRCA
		HLVT_DRFP
DS	Focused, selective, and sustained attention and visual perception	DigSim

MMSE: Mini-Mental State Examination; BTA: Attentional Span or Brief Test of Attention; TMT: Trail Making Test; HLVT: Hopkins Verbal Learning Test; DS: Digit Symbol Substitution Test; MMSE_sc: Score of MMSE; AtentL: total score in Form L in BTA; AtentN: total score in Form N in BTA; TMT_PartA: subject’s score in TMT Part A; TMT_PartB: subject’s score in TMT Part B; HLVT_T1: score in trial 1 in HLVT; HLVT_T2: score in trial 2 in HLVT; HLVT_T3: score in trial 3 in HLVT; HLVT_TR: score in the trial executed 20 minutes later after the reading in HLVT; HLVT_DRCA: delayed recall correct answers in HLVT; HLVT_DRFP: delayed recall false positives in HLVT; DigSim: subject’s score in DS.

Cognitive variables listed in Table 1 have normative data for the Cuban adult population obtained as part of an international collaborative study [35–38].

### Cognitive indexes

The four cognitive indexes were computed as follows:

1- MMSE z-score: The MMSE score (MMSE_sc) was transformed into the MMSE z-score (*z*_*iMMSE*_) using Eq 2.


$$z_{iMMSE}={(v}_{iMMSE}-\mu_{MMSE})/\sigma_{MMSE}$$ (2)

Where:

*v*_*iMMSE*_: MMSE score for the subject *i*

*μ*_*MMSE*_: mean value of the MMSE score in the population of normal subjects, adjusted for age and educational level

*σ*_*MMSE*_: standard deviation of the MMSE score in the population of normal subjects, adjusted for age and educational level

2- ZSum: This index was defined as the sum of the z-scores for the 12 cognitive variables, and was calculated using Eq 3.


$${ZSum}_{i}=\sum_{j=1}^{12}z_{ij}$$ (3)

Where:

*z*_*ij*_: is the z-score of the cognitive variable *j* for subject *i*, calculated using Eq 4:

$$z_{ij}={(v}_{ij}-\mu_{j})/\sigma_{j}$$ (4)

*μ*_*j*_: mean value of the cognitive variable *j* in the population of normal subjects

*σ*_*j*_: standard deviation of cognitive variable *j* in the population of normal subjects

These two population parameters were calculated using normative regression functions of age- and educational level-dependent mean values and standard deviations obtained from 306 normal subjects aged between 18 and 90 years, corresponding to the normative data for the Cuban adult population used in the collaborative study mentioned earlier [35–38].

3- PCCog: PCA was applied to the z-score vector of the 12 cognitive variables, and the scores of the first principal component (explaining the largest proportion of variance) were retained. PCA is commonly used for dimensionality reduction [19], and the first principal component of a set of psychological tasks is often used to assess Spearman’s G factor of (fluid) intelligence [39].

4-MDCog: This index was calculated using the Mahalanobis distance to measure deviations from normative data, considering the correlations between the variables, using Eq 5.


$${MDCog}_{i}^{2}=Z_{i}^{\prime }\sum^{-1}Z_{i}$$ (5)

Where:

*Z*_*i*_: vector with the scores of the 12 cognitive variables for subject *i*, adjusted for age and educational level

∑^−1^: represents the covariance matrix of the population norm

The Mahalanobis distance has been previously applied as a spatial measurement of the participant´s cognitive profile deviation from the average profile of the population [20,21]. In this case, normative data for the Cuban population in the five cognitive were used [35–38]. This new cognitive index provides an objective and continuous measure of the subject’s cognitive state, taking into consideration the correlation between the results in each neuropsychological test.

### Analysis

PCA was applied to the original set of 96 STGF to create a new set of features called PCA-STGF. This was done because the original features were likely highly correlated. The loadings and scores of the principal components, which explained over 95% of the variance, were retained. The loadings were used to assess the contribution of each STGF to the different walking tasks, while the scores were used to examine the relationship with cognitive variables. PCA was also applied to the cognitive variables to obtain the summary cognitive index PCCog, as explained in the previous section.

To investigate the relationship between the selected components of PCA-STG (independent variables) and the 15 dependent variables (four summary cognitive indexes, and the 11 *z*-scores of the cognitive variables), multiple linear regressions were performed. The adjusted proportion of variance (adjusted-R^2^) that the independent variables could predict the dependent variables was calculated for each regression. The confidence intervals for adjusted-R^2^ were estimated by bootstrapping the data and repeating the regressions 1000 times. The values of F statistic and probability for F-test were also considered when comparing the results of the multiple linear regressions. The F statistic tests whether the model fits significantly better than a degenerate model consisting of only a constant term, while the probability indicates if the model is significant or not. The Frisch-Waugh-Lovell theorem was used to visualize the fit of the dependent variables versus the adjusted predictor variable values in the best models [40, 41]. The best models were determined by selecting those with the minimum difference between their adjusted-R^2^ and the average of adjusted-R^2^ for all models obtained through bootstrapping. All variables used in the multiple linear regressions were adjusted for age and educational level.

All data analysis was conducted offline using MATLAB® (Mathworks Inc.).

## Results

A total of 125 participants completed all requirements and took part in the study. They were divided into two groups: healthy adults and adults with MCI. The division into groups was done using MDCog-based k-means clustering (1000 replicates). The healthy group was further divided by age, resulting in three groups: young adults (n = 40, age: 27.65 ± 4.15 years, 50% women), healthy older adults (n = 62, age: 72.23 ± 6.6 years, 59.68% women), and MCI older adults (n = 23, age: 76 ± 7.43 years, 69.57% women). The demographic data of the selected sample are presented in Table 2.

**Table 2:** Demographic data of the sample.

	Young adults	Healthy older adults	MCI older adults
**N**	40	62	23
**Age (years)**	27.65 ± 4.14 [22–38]	72.23 ± 6.59 [60–88]	76 ± 7.43 [61–87]
**Sex (% women)**	20 (50)	37 (59.7)	16 (69.5)
**MMSE score**	29.53 ± 0.88 [27–30]	28.39 ± 1.62 [24–30]	22.91 ± 2.84 [18–29]
**Education**	Primary: 0 Middle: 0 Secondary: 6 Tertiary: 34	Primary: 1 Middle: 11 Secondary:30 Tertiary: 20	Primary: 10 Middle: 3 Secondary: 5 Tertiary: 5

Values are presented as mean ± STD.

The range is given in square brackets.

The four summary cognitive indexes were calculated for the 125 participants. The distribution of the values for these indexes are presented in S2 Fig in S1 File.

PCA was applied to the original set of 96 STGF, and it confirmed the high level of redundancy between the features. The first seven principal components of PCA-STGF explained over 95% of the variance and were retained for further analyses (highlighted with red dots in S3 Fig in S1 File), instead the original features. The loadings of the first principal component, which represents the contribution of each STGF to the component, are shown in S4 Fig in S1 File. They were reshaped into four columns of 16 features, with each column representing one walking task. It can be observed that the contribution of each STGF was similar across all four walking tasks. The coefficients corresponding to gait variability features ranged from 0.07 to 0.1, while the features related to the description of the gait cycle had coefficients around 0.1. The coefficients for Cd, RMS value, StepLg, and GS were around -0.1 in all tasks. Since the first PCA component explained 68.79% of the variance, it suggests that some walking conditions could be eliminated in the experiment without losing much information, leading to a shorter examination time.

**Fig 2:**
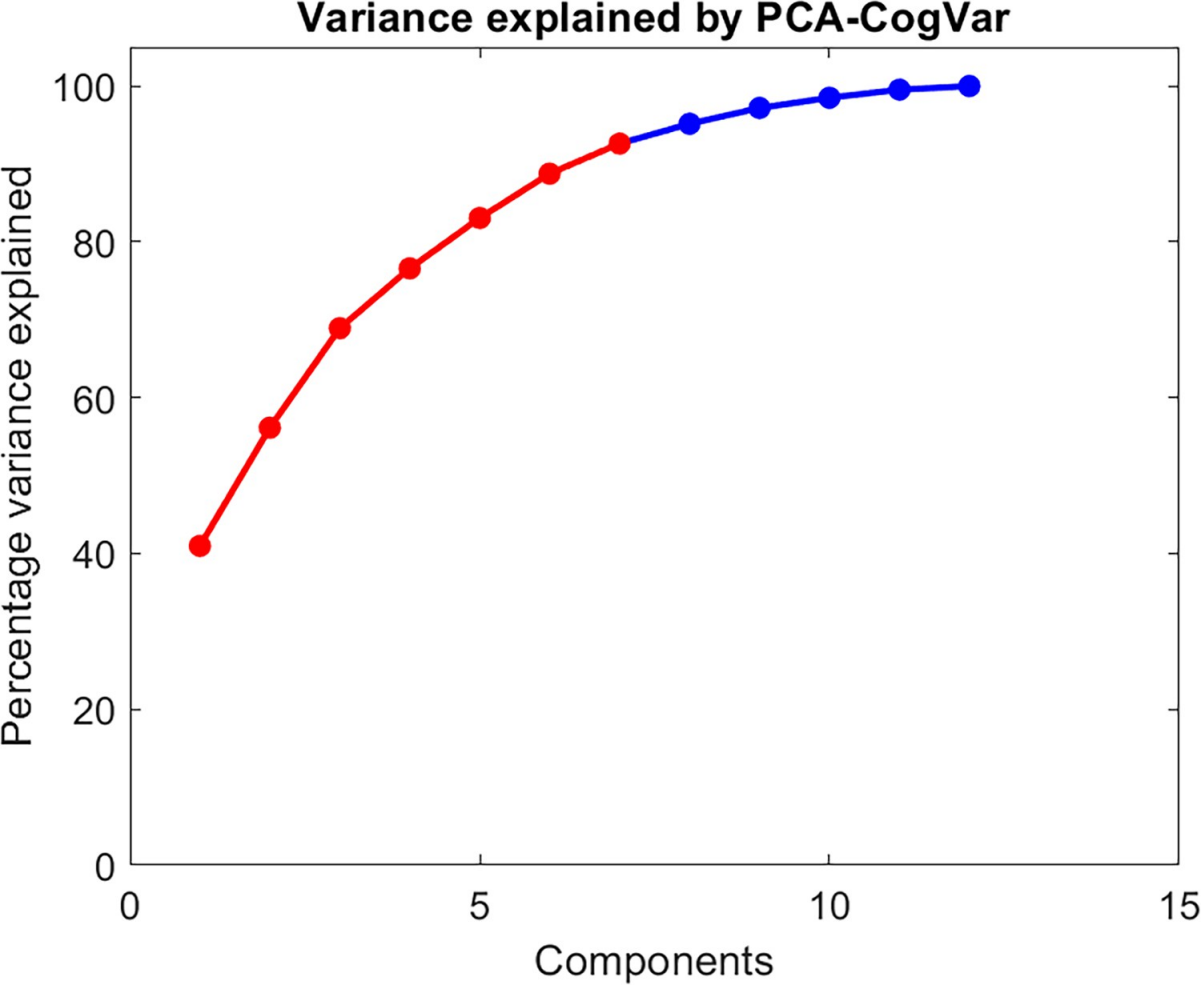
The cumulative sum of the variance accounted for the 12 principal components of the cognitive variables.

After applying PCA to cognitive variables, the cumulative sum of the variance accounted for the new 12 principal components (PCA-CogVar) is shown in Fig 2. The first seven components explained over 95% of the variance and are highlighted with red dots in the figure. The first component accounted for the highest percentage of variance (40.98%), and its scores were retained as the PCCog summary cognitive index.

Table 3 summarizes the estimated performance of the multiple linear regressions models for predicting the summary cognitive indexes from PCA-STGF.

**Table 3:** Performance of the multiple linear regression models for the summary cognitive indexes.

Variable	Adjusted-R^2^	F	p	CI
MMSE z-score	0.0248	1.4848	0.3168	[-0.0307–0.1001]
ZSum	0.0080	1.1699	0.4454	[-0.0393–0.0746]
PCCog	0.0033	1.0752	0.4611	[-0.0357–0.0585]
MDCog	0.4445	15.8402	0.0000*	[0.3610–0.5753]

* Statistically significant

MMSE z-score: the z-score of Mini-Mental State Examination; ZSum: the sum of the absolute z-scores of the patient’s neuropsychological measures from a population norm; PCCog: the patient’s scores for the first principal component of the neuropsychological test scores; MDCog: the Mahalanobis distance of each patient’s score from a population norm; F: F statistic; p: probability; CI: confidence interval for adjusted-R^2^.

The multiple linear regression model for MDCog was the only one that showed statistical significance, with the highest value of the adjusted-R^2^ estimation. Table 3 also presents the confidence intervals for adjusted-R^2^, which were estimated by bootstrapping the regression 1000 times. The PCA-STGF was able to predict a larger proportion of the variance for MDCog compare to the other summary cognitive indexes.

**Fig 3:**
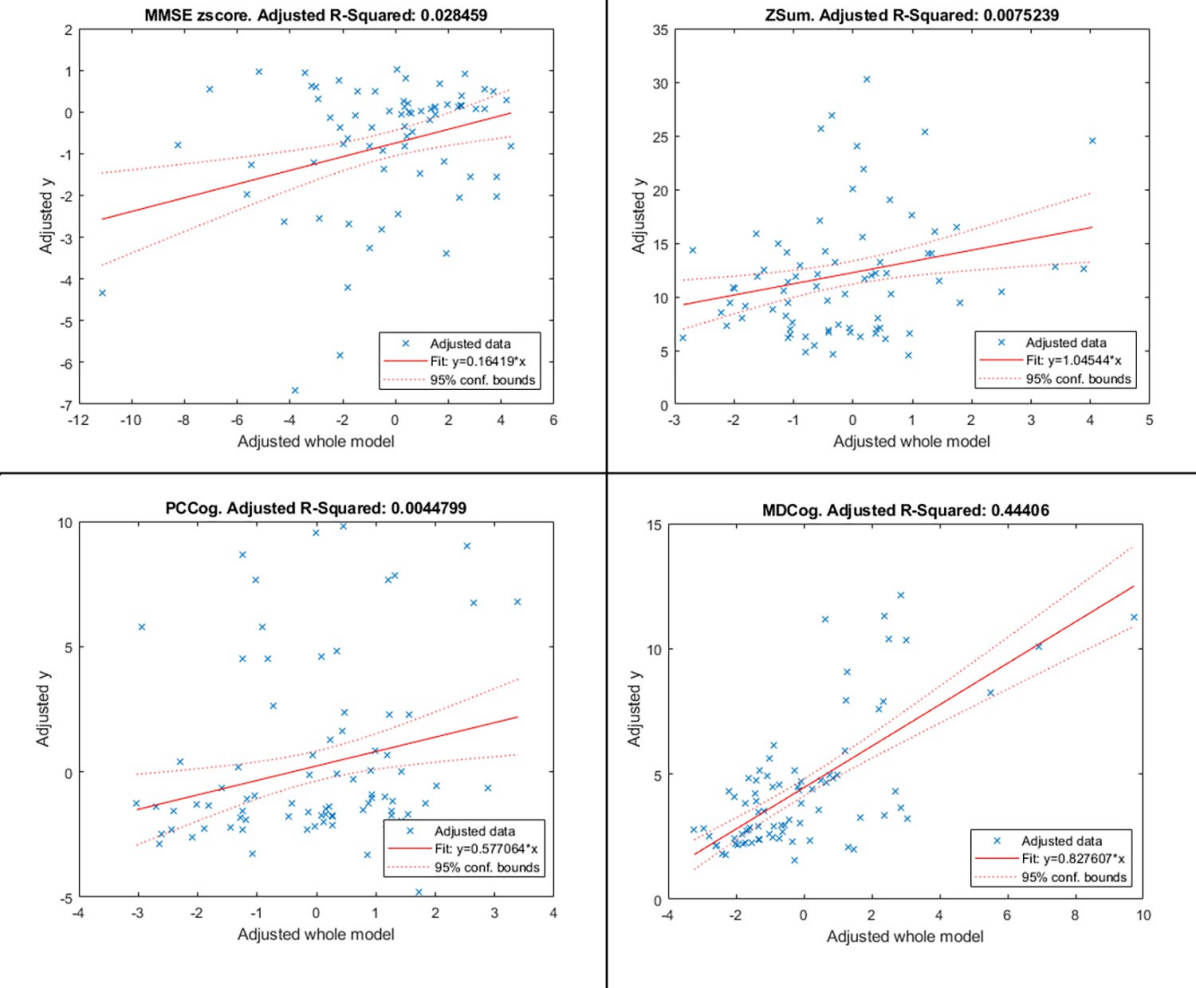
Plots of the best models for the four summary cognitive indexes.

Fig 3 show the plots of the best models for each summary cognitive index. These plots help to visualize the significance of the model by presenting a scatter plot of adjusted response values (y-axis) against adjusted predictor variable values (x-axis), representing the model as a whole. The fitted line for adjusted response values as a function of adjusted predictor variable values, along with the 95% confidence bounds of the fitted line, are also shown.

Overall, Fig 3 confirms that the MDCog model is the most significant one, as it has the highest slope, and a horizontal line cannot be fitted between the confidence bounds.

New multiple linear regressions with bootstrapping were applied to assess the predictability of the individual cognitive variables z-scores, using the same independent variables (the first seven components of PCA-STGF). Table 4 presents the performance of the models obtained in these multiple linear regressions.

**Table 4:** Performance of the multiple linear regression models for the z-scores of the individual cognitive variables.

Variable	Adjusted-R^2^	F	p	CI
AtentL	0.0483	1.9484	0.1937	[-0.0222–0.1329]
AtentN	0.0693	2.3732	0.1084	[-0.0018–0.1581]
TMT_PartA	0.0172	1.3310	0.3426	[-0.0269–0.0796]
TMT_PartB	0.0108	1.2179	0.4083	[-0.0362–0.0786]
HLVT_T1	0.0431	1.8305	0.1901	[-0.0145–0.1143]
HLVT_T2	0.0679	2.3457	0.1115	[-0.0041–0.1577]
HLVT_T3	0.0664	2.3106	0.1071	[-0.0009–0.1538]
HLVT_TR	0.0365	1.7106	0.2413	[-0.0221–0.1205]
HLVT_DRCA	0.0344	1.6726	0.2638	[-0.0266–0.1183]
HLVT_DRFP	0.0009	1.0292	0.4739	[-0.0366–0.485]
DigSim	0.0310	1.6072	0.2871	[-0.0315–0.1099]

AtentL: total score in Form L in Attentional Span or Brief Test of Attention; AtentN: total score in Form N in Attentional Span or Brief Test of Attention; TMT_PartA: subject’s score in Trail Making Test Part A; TMT_PartB: subject’s score in Trail Making Test Part B; HLVT_T1: score in trial 1 in Hopkins Verbal Learning Test; HLVT_T2: score in trial 2 in Hopkins Verbal Learning Test; HLVT_T3: score in trial 3 in Hopkins Verbal Learning Test; HLVT_TR: score in the trial executed 20 minutes later after the reading in Hopkins Verbal Learning Test; HLVT_DRCA: delayed recall correct answers in Hopkins Verbal Learning Test; HLVT_DRFP: delayed recall false positives in Hopkins Verbal Learning Test; DigSim: subject’s score in Hopkins Verbal Learning Test; F: F statistic; p: probability; CI: confidence interval for adjusted-R^2^.

None of the z-scores of the cognitive variables were statistically significant, as shown in Table 4. The adjusted-R^2^ values were smaller compare to the value obtained for MDCog.

## Discussion

In this study, we examined four cognitive summary indexes to determine which one could be most accurately predicted by STGF. Due to significant redundancy between the STGF of different walking tasks, we utilized the first seven components that accounted for 95% of the variance. MDCog demonstrated the closest association with gait features, with gait parameters accounting for more variance in MDCog compare to other summary indexes or individual cognitive scores. This summary cognitive index served as a quantitative measure of the cognitive trait of any subject, from young adults to older adults with cognitive impairment.

Two possible explanations exist for the superiority of MDCog. First, combining indicators of ability in multiple areas into a global measure can help compensate for measurement noise in each test and provide an overall index of the subject’s cognitive function [42]. Second, it has been long known from factor analysis of multiple psychological tests that a general latent variable (Spearman’s G factor) explains a large proportion of the variance between individuals [43, 44]. This G factor has been associated to fluid intelligence [39], effectiveness in executive function tasks [45], and has been shown to decline with aging [44]. Our MDCog could utilized information related to this factor, which taps essential processes needed to coordinate specific behaviors and manage multitasking. At the same time, it retains information about specific processes such as memory and attention.

The lower performance of the other summary cognitive indexes could be due to several causes. Although the MMSE is a widely used global cognition index, its use has been criticized since its scores depend on the educational level of the subjects and whether they are illiterate or not [9]. Moreover, its sensitivity in discriminating MCI patients from those with normal cognitive aging is inadequate [8, 9, 46]. On the other hand, the sum of the z-scores does not take into account the sign of the score of each variable or the correlation between them. Thus, a large pathological deviation on one variable will be added to a better-than-average performance, overestimating the real deterioration, and summing the z-values of the highly correlated variables will also exaggerate scored anomaly.

Regarding the application of PCA to the cognitive variables, we used only the first component, as customary in the estimation of fluid intelligence. In our case, it explained only 40.98% of the variance in the cognitive variables, leaving 59.02% unexplained. Thus, it discards much information, which is a disadvantage of this index compared to MDCog. This Spearman’s factor G by itself does not have a strong association with gait. MDCog considers the correlation between the results in each neuropsychological test and the distance of each subject from normative data. These factors, together with those stated above, likely explain the better results obtained with this index.

Many of the STGF used in this study describe the gait cycle, so there is a high correlation between them. This redundancy in the data has been recognized as a problem in gait analysis by other authors [47]. To address this issue, we applied PCA, a statistical procedure commonly used in gait analysis studies [44–49], to convert the correlated features into a smaller set of linearly uncorrelated components [50]. We found that the first seven principal components explained 95% of the variance. Although the use of the PCA could mask the contribution of the original STGF to the predictability of the evaluated cognitive indexes, we observed that some STGF with the highest values (Cd, RMS value, StepLg, and GS) have been previously reported as good predictors for MCI [8, 21], as supported by the assessment of the first principal component coefficients.

This study provides insights into the relationship between the cognitive status and STGF, suggesting potential clinical applications. Neuropsychology provides many measured variables, but clinicians often need to subjectively synthesize this data to form a clinical impression that is either nominal or ordinal. By predicting the degree of cognitive impairment, STGF could be useful for clinicians and help reduce errors in subjective judgments.

One limitation of this study is the smaller sample size of older adults with cognitive impairment compared to the healthy sample. Additionally, both samples were not fully paired by sex and age, which is another limitation. Future studies should aim to increase sample size and better stratify by age. It is also recommended to further investigate the relationship between STGF domains and cognitive domains further, possibly through canonical correlation analysis.

## Conclusions

In conclusion, our study confirms the utility of MDCog as a reliable and quantitative measure of cognitive function. MDCog, which is based on the Mahalanobis distance from each subject’s cognitive measure to the population norm and the interaction among these factors, demonstrated a significant association with the STGF. It shows the highest value of the proportion of the variance that can be explained by the predictor variables. These findings suggest that MDCog may serve as an objective measure of cognitive function in various populations and could be used as a useful tool in studying the relationship between gait patterns and cognition.

## Supporting information

S1 File (DOCX)
