# Supplementary material for: Relationship between gait parameters and cognitive indexes in adult aging
Source: PLoS One. 2023 Sep 21;18(9):e0291963. doi: 10.1371/journal.pone.0291963 (PMC10513272; doi:10.1371/journal.pone.0291963)
Supplement: S1 File — (DOCX) [file pone.0291963.s001.docx]

Supporting information

# Supporting Figures

**Recruitment steps:**

Informed consent was obtained from all the participants and from the doctors of the health institutions where the experiment was carried out.

A questionnaire, called Katz index, was applied to all the older adults for identifying the degree of independence to carry out the basic activities of daily life. Subjects with a Katz index less than four were excluded.

A questionnaire was applied to carry out a clinical evaluation that included pathological history, associated comorbidities, toxic habits, as well as gait problems.

The participants underwent a neurological and physical examination to explore disorders that could affect gait. The exam included aspects such as orientation, muscle strength, eye tracking, deep sensation, superficial sensation, tendon reflexes, and coordination and balance. Those participants evaluated as Poor in this test were excluded.

A battery of five neuropsychological tests was applied to determine the cognitive state of the participants, for identifying the healthy participants, and the participants with Mild Cognitive Impairment (MCI). The battery was composed of: 1) Mini-Mental State Examination (MMSE), as a global index of cognition; 2) Attentional Span or Brief Attention Test (BTA), as a measure of auditory divided attention; 3) Trail Making Test (TMT), parts A and B, to assess attention, visuospatial abilities, mental flexibility, and executive functions; 4) Hopkins Verbal Learning Test (HLVT) for memory assessment, including immediate recognition and delayed recall; and 5) Digit Symbol Substitution Test (DS), for focused, selective and sustained attention, as well as visual perception. Those subjects with an MMSE score below 20 and/or with poor performance in the rest of the tests were excluded.

The gait patterns were recorded while the participants executed four walking tasks in an obstacle-free and flat environment. Each participant cover 40 m in a straight line, 20 m in each direction, as is shown in Supplementary Figure 2. The walking tasks were: 1) walking freely at a comfortable self-chosen speed (NormalW); 2) walking at a comfortable self-chosen speed while simultaneously counting their steps, an easy cognitive task (EasyD); 3) walking at a comfortable self-chosen speed while simultaneously counting backward from 100, a hard cognitive task (HardD); and 4) walking as fast as possible without running (FastW).


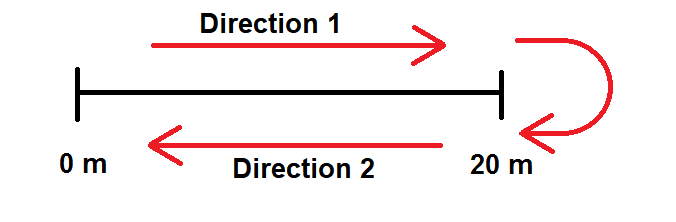


**S1 Fig. Experimental environment.**

In all steps, the participants with missing data were excluded from the sample.

**Summary cognitive indexes**


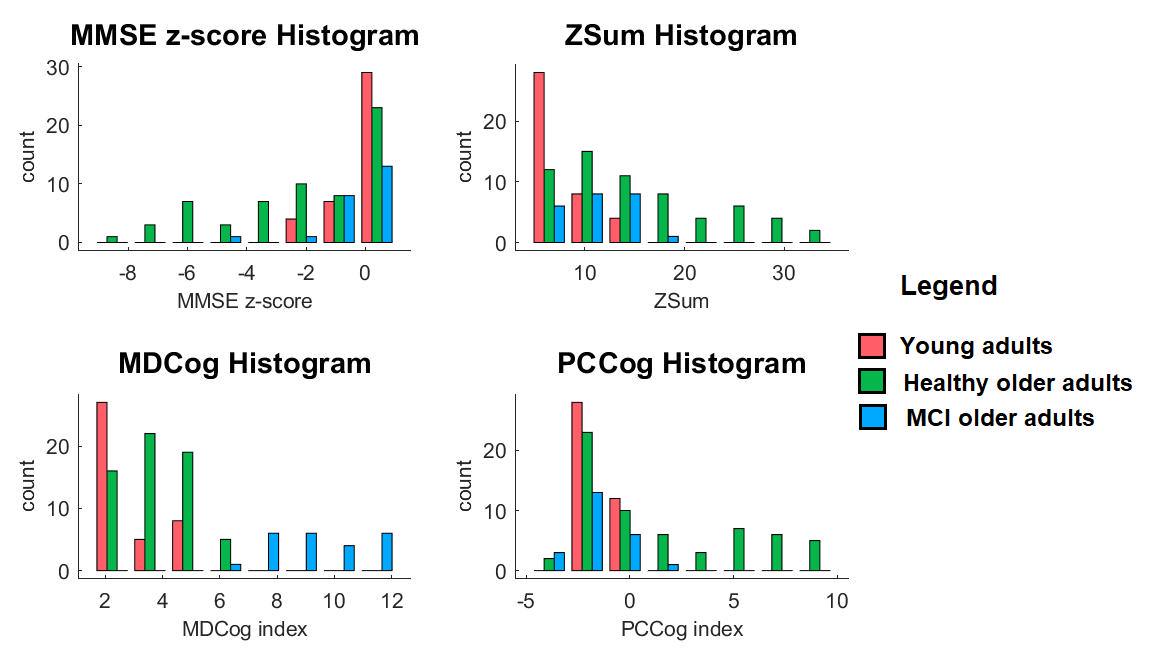


**S2 Fig. Histograms of the four summary cognitive indexes corresponding to the three groups: young adults, healthy older adults and older adults with Mild Cognitive Impairment (MCI older adults).**

**
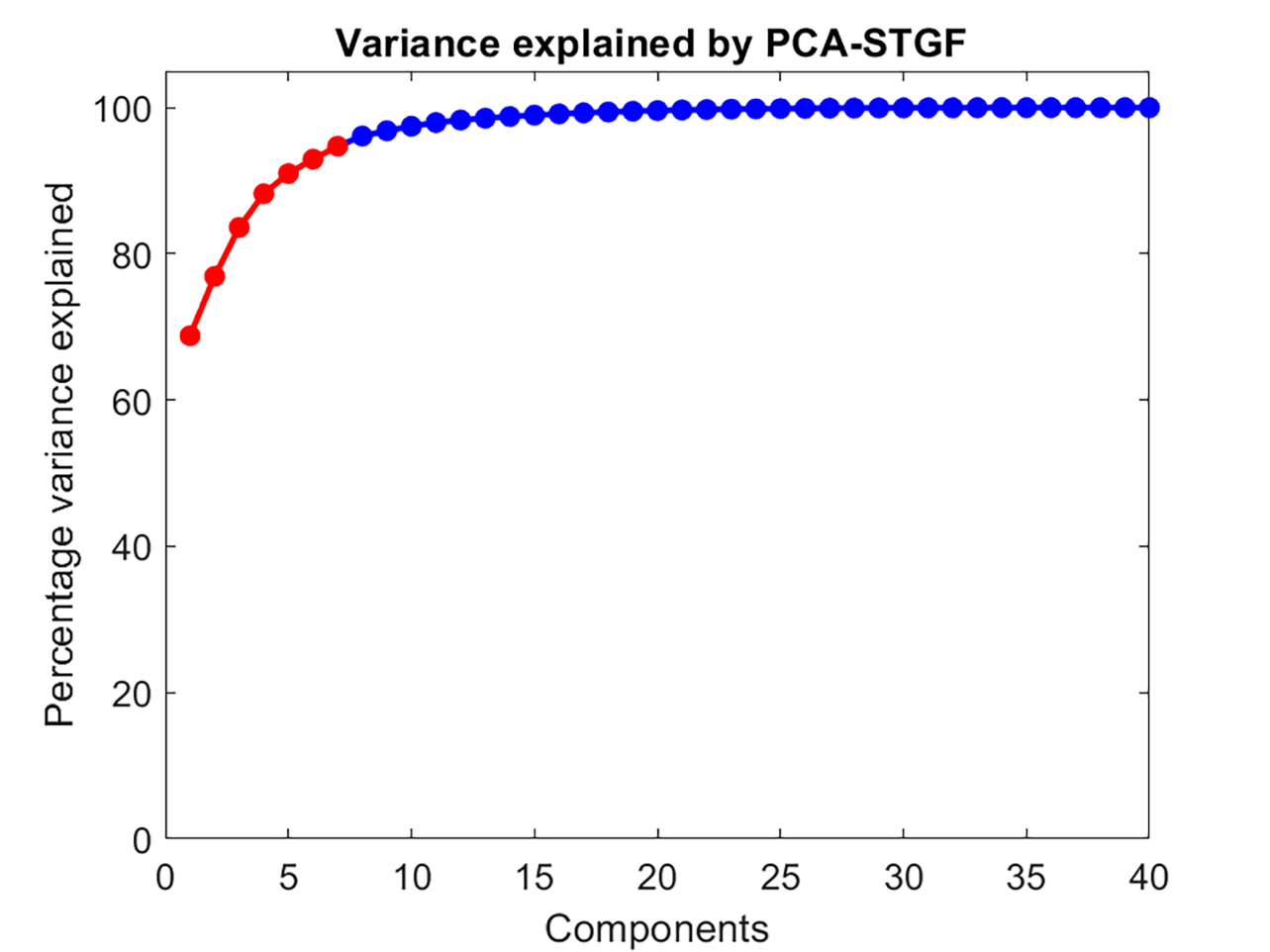
**

**S3 Fig. The cumulative sum of the variance accounted for the first 40 principal components of the new set of features PCA-STGF.**


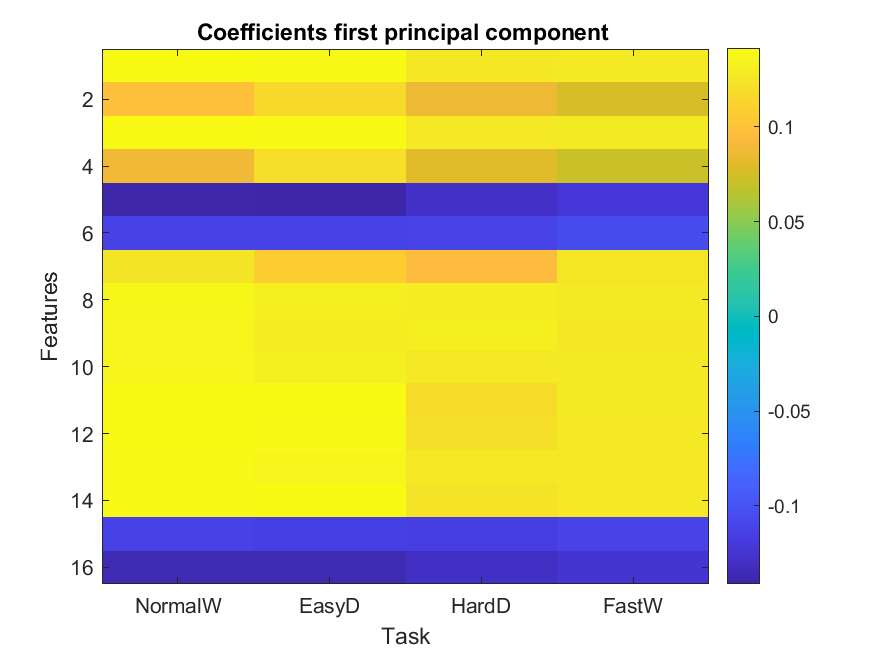


**S4 Fig. Contribution of the 16 STGF in each task to the first principal component of PCA-STGF.**

# Supporting Tables

**S1 Table. Sample composition.**

| **Institution** | **Participants included** | **Participants excluded** | **Total evaluated** |
| --- | --- | --- | --- |
| Cuban Center for Neuroscience* | 56 | 20 | 76 |
| Health areas** | 37 | 9 | 46 |
| Nursing homes** | 14 | 21 | 35 |
| Grandparent´s homes** | 18 | 15 | 33 |
| Total | 125 | 65 | 190 |

* 40 young adults and 16 older adults

** Older adults
